# Mobile Toolbox sequences task: development and validation of a remote, smartphone-based working memory test

**DOI:** 10.3389/fpsyg.2024.1497816

**Published:** 2025-01-22

**Authors:** Jerry Slotkin, Aaron J. Kaat, Stephanie Ruth Young, Elizabeth M. Dworak, Miriam A. Novack, Yusuke Shono, Hubert Adam, Cindy J. Nowinski, Sarah Pila, Zahra Hosseinian, Maria Varela Diaz, Anyelo Almonte-Correa, Keith Alperin, Monica R. Camacho, Bernard Landavazo, Rachel L. Nosheny, Michael W. Weiner, Richard C. Gershon

**Affiliations:** ^1^Center for Health Assessment Research and Translation, University of Delaware, Newark, DE, United States; ^2^Department of Medical Social Sciences, Northwestern University Feinberg School of Medicine, Chicago, IL, United States; ^3^School of Community and Global Health, Claremont Graduate University, Claremont, CA, United States; ^4^Helium Foot Software, Inc, Chicago, IL, United States; ^5^University of California, San Francisco, San Francisco, CA, United States; ^6^Northern California Institute for Research and Education, San Francisco Veteran's Administration Medical Center, San Francisco, CA, United States

**Keywords:** cognition, working memory, mobile assessment, NIH Toolbox, validation

## Abstract

**Objective:**

The ability to assess cognitive skills remotely is increasing with the widespread use and availability of smartphones. The Mobile Toolbox (MTB) is a measurement system that includes Sequences, a new measure of working memory designed specifically for smartphones. This study describes the development of Sequences and presents the studies conducted to evaluate its psychometric properties.

**Methods:**

We developed a new measure of working memory that can be self-administered remotely using an iOS or Android smartphone. In Sequences, a series of numbers and letters are shown on the screen one at a time, and the participant must first tap the letters they see in alphabetical order, followed by tapping the numbers in ascending numerical order. The Sequences measure was evaluated for usability and feasibility across two pilot studies and then assessed in this validation study (which included a total sample size of *N* = 1,246). Psychometric properties of the new measure were evaluated in three studies involving participants aged 18–90 years. In Study 1 (*N* = 92), participants completed MTB measures in a laboratory setting. They were also administered both an equivalent NIH Toolbox (NIHTB) measure along with external measures of similar constructs. In Study 2 (*N* = 1,007), participants were administered NIHTB measures in the laboratory and then completed MTB measures remotely on their own devices. In Study 3 (*N* = 147), participants completed MTB measures twice, remotely on their own devices, with a 2–week interval between sessions.

**Results:**

Sequences exhibited moderately high correlations with a comparable NIHTB test and external measures of a similar construct, while exhibiting a lower correlation with an unrelated test, as hypothesized. Internal consistency was high, but test-retest reliability was moderate. When controlling for age, phone operating system (iOS vs. Android) and sex assigned at birth did not significantly impact performance; however, there was a significant difference between individuals who completed college and those with a high school education or lower.

**Conclusion:**

The results support the validity of Sequences as a measure of working memory for remote self-administered use. The internal consistency was strong, with moderate test-retest reliability that is likely a function of the test's unproctored self-administration method. The findings suggest that Sequences is appropriate for use with adults aged 18–90 years in remote self-administered designs that focus on group results.

## Introduction

Accurate, economical, and efficient measures of working memory are critical in both neuropsychological research and clinical contexts. Working memory is commonly defined as a cognitive process in which information is stored and manipulated over a brief period of time. This includes holding information in short-term memory, manipulating the information, and then holding the products of that manipulation in the same short-term memory (Baddeley, 1992). Working memory is involved in many activities of daily living, such as real-world decision-making (Kleider-Offutt et al., 2016), driving (Watson et al., 2016), academic and vocational tasks (Bosco et al., 2015; Aronen et al., 2005), and recreational activities (Furley and Wood, 2016). Moreover, working memory deficits are common across many neurological and developmental disorders and brain injuries (Cai et al., 2021). Measuring working memory abilities can be of particular importance for older adults, as its decline can be associated not only with the natural aging process but also with pathological cognitive decline (Kirova et al., 2015).

Sequencing tasks that involve both storage and manipulation of information are among the most common tasks to measure working memory capacity (Conway et al., 2005). In the Baddeley's model framework (Baddeley, 1992), sequencing tasks engage the phonological loop or visual-spatial sketchpad to hold auditory or visual information in temporary storage, while the central executive system is used to organize the information in a particular way (e.g., largest to smallest or alphabetically). More-complex sequencing tasks often require the central executive system to categorize multiple types of information before ordering them (e.g., grouping by numbers or letters). For example, the List Sorting Working Memory Test (LSWMT, 10) was included in the National Institutes of Health Toolbox^®^ for Assessment of Neurological and Behavioral Function Cognition Battery (NIHTB-CB) for more than a decade. It has also been used in hundreds of research studies (Fox et al., 2022). LSWMT is a complex sequencing task in which examinees are presented with two categories of items (food and animals) both visually and auditorily. Examinees are then asked to sort the items by category and arrange them in order of size, providing their responses orally.

Building on the success of the NIHTB, the NIH funded the Mobile Toolbox (MTB)—a comprehensive assessment library aimed at improving measurement tools to monitor cognitive functioning over time and to identify pathological cognitive decline (Gershon et al., 2022). The MTB enables researchers to design their own studies and create customized test batteries that participants can complete independently on their smartphones. Remote data collection on smartphones offers an economical way to collect large-scale longitudinal data from diverse and often difficult-to-access populations (Hensen et al., 2021; Tiersma et al., 2022). The cognitive constructs measured in the MTB library were modeled based on the NIHTB-CB (Weintraub et al., 2013). While some measures are direct analogs of the NIHTB-CB measures adapted for mobile devices and unproctored administration (e.g., MTB Arrows/NIHTB Flanker) (Novack et al., 2024), other constructs, including working memory, required the creation of *de novo* tests to assess performance in a self-administered, smartphone format. In response to this need, we created the Sequences test. In this test, examinees are visually presented with a series of numbers and letters, and they are asked to arrange the letters in alphabetical order and the numbers in ascending order using a smartphone keyboard. Here, we describe the development of Sequences, a new working memory measure in the MTB library, and examine its psychometric properties across three studies.

A small number of studies have investigated the feasibility and utility of smartphone-based working memory tasks in research settings (Hakun et al., 2023; Nicosia et al., 2023; Weizenbaum et al., 2022). Initial evidence supports the assessment of working memory on a smartphone device, with small to moderate convergent validity reported. These studies have mostly utilized measures that assess other aspects of working memory, such as span, short-term memory after interference, or spatial memory, whereas the new Sequences test involves more complex mental manipulation of the presented stimuli, utilizing a more traditional working memory assessment paradigm.

## Materials and methods

### Measure development

The goal of the Sequences measure is to create a working memory test that is optimized for self-administration on a smartphone. To achieve this goal, we identified the following parameters for the task that guided its development: (1) The task should minimize reliance on audio delivery of stimuli, as this can introduce variability and errors based on individual smartphone volume settings and background noise in the individual's testing environment. (2) The task should not rely on oral responses, but rather responses should be entered directly into the app interface using the touchscreen. (3) Visual stimuli should be simple to be viewed clearly on a smartphone screen. (4) The task should be designed to be completed by participants in no more than 7 min (and ideally in under 5 min), while assessing a broad range of ability. In the MTB Sequences task, participants are shown a series of numbers and letters displayed sequentially on the screen, one at a time, every second. They must first enter what they see by tapping the letters in alphabetical order and then enter the numbers from smallest to largest using a customized on-screen keyboard to submit their responses.

Prior to any development, Sequences underwent an iterative design and specification process to ensure its utility for remote self-administration across multiple smartphone platforms. For memory tests such as Sequences, we focused on several key factors: the time between stimulus presentation and retrieval, expected behavior during and after an interruption, and the potential risks related to the size and selection of response options on smartphone screens. We conducted pilot studies, which are described in detail in our Supplementary material, to investigate the feasibility and stimuli selection before finalizing the measure for broader data collection and use. Finally, Sequences underwent user interface (UI) and user experience (UX) testing to evaluate the interface, presentation, and response modality.

### Pilot testing and usability study

Two separate pilot studies were conducted to support the development of Sequences and assess the feasibility of self-administration on a personal smartphone in a remote context. More details about the pilot studies are available in our Supplementary material. After conducting feasibility trials with the most common size of iPhone (5.8″ screen size at the time), it was determined that a maximum of nine numbers and nine letters could be displayed on the screen simultaneously for participant responses while minimizing participant data entry error, which could confound a working memory test. Data from Pilot Study 1 showed that there were significant floor effects (Terwee et al., 2007), as approximately one-quarter of the respondents did not answer any items correctly on the forms. Changes were implemented to incorporate easier content and improve task comprehension for Pilot Study 2. These changes substantially improved performance, floor effects were resolved, and no ceiling effects were evident in Pilot Study 2.

In addition to the pilot studies, we employed a qualitative expert to elicit feedback from naïve users on the layout of the test and the ease of use on an iPhone. Our adaptation and development processes included user experience testing for MTB. A skilled qualitative researcher and UI/UX design expert conducted individual sessions with participants who were naïve to the test development process. Participants evaluated the Pilot Study 1 version of Sequences in the MTB app, providing input regarding whether they could identify what the test was supposed to assess, how easy or hard it was to complete the measure, and the “look and feel” of the measure. In cases where participant feedback indicated that design changes were necessary, the UI designer collaborated with the scientific team to ensure that the recommended changes were appropriate and would not compromise the assessment of the construct. The UI/UX review for Sequences included challenges in understanding the task and difficulties with visual discrimination of the letters. This feedback led to modifications discussed earlier for the Pilot Study 2 version of the app.

**Figure 1:**
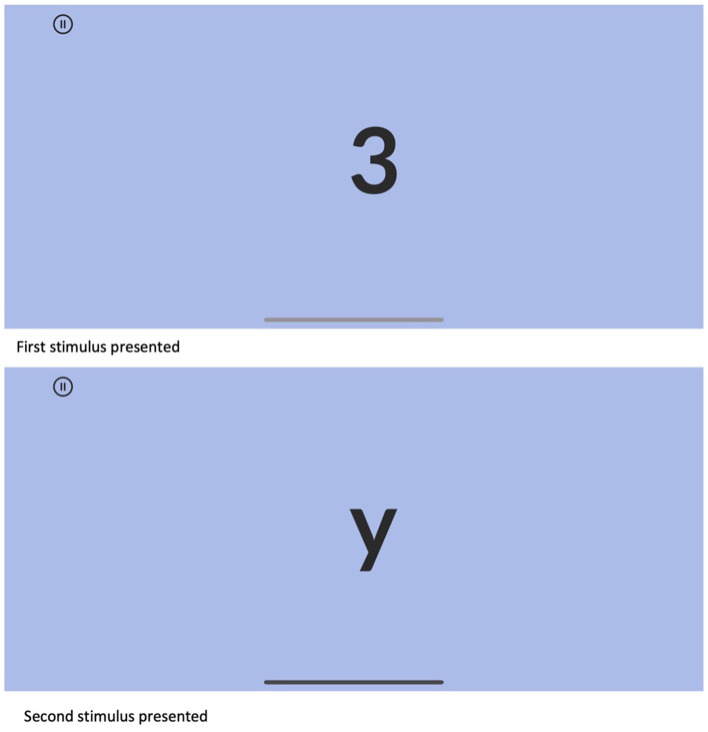
Example item presentation, sequence length 2.

**Figure 2:**
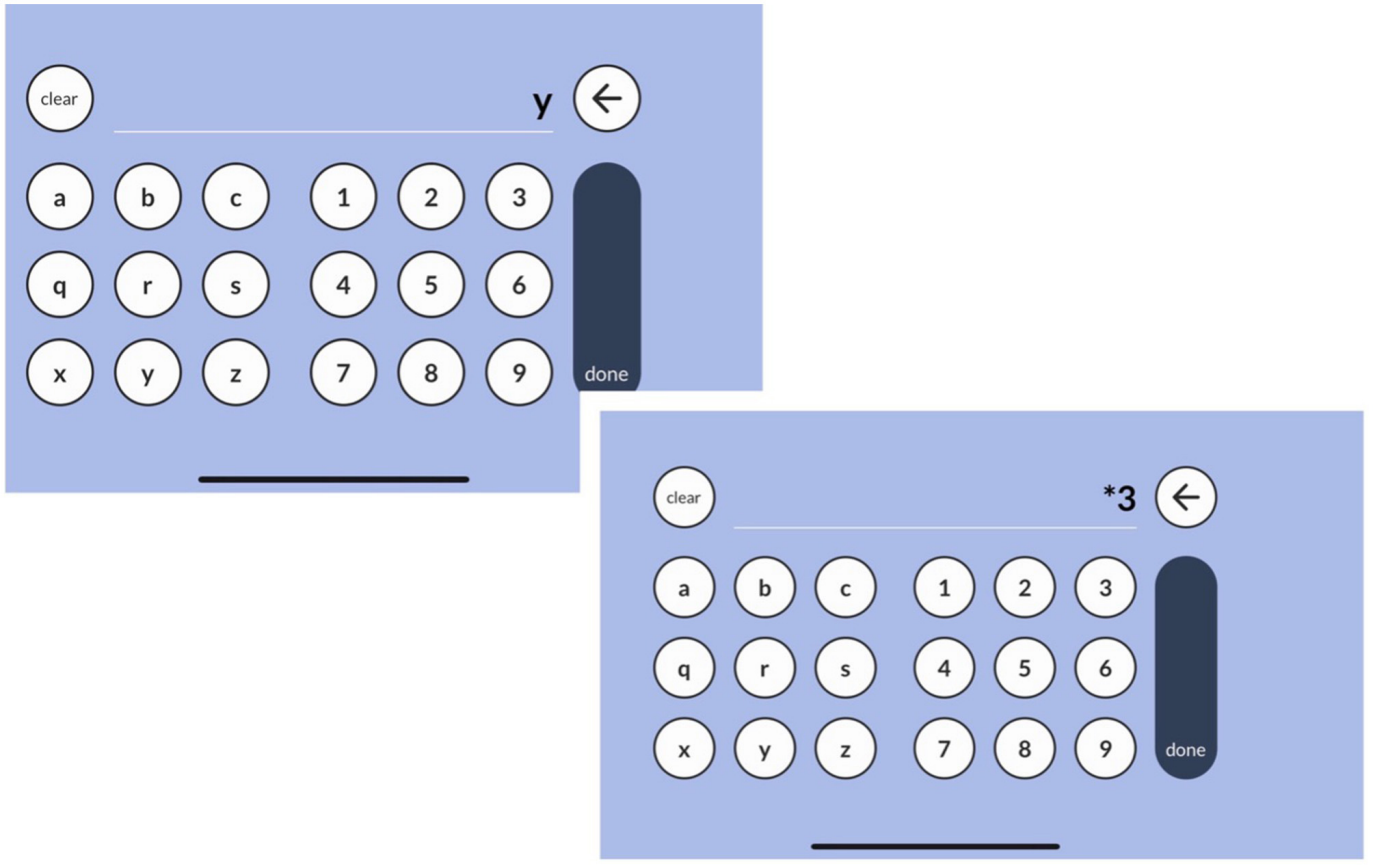
Response entry screens for example item.

Based on the aggregation of qualitative and quantitative data gathered from both pilot studies and usability tests, Sequences appeared to be ready for formal validation studies. The final presentation modality for the version used in the validation studies, which are the focus of this study, included the following: (1) three simple instruction screens, with animated examples showing participants how to complete the test, along with two practice trials that provide corrective feedback; (2) content adjustments, including one set of trials at a sequence length of two, and two sets of trials at a sequence length of three; (3) lowercase letters grouped in distinct sets of three rather than being continuous (specifically, a-b-c-q-r-s-x-y-z); (4) a discontinue rule requiring all three items within a sequence length set to be answered incorrectly. An example of an item presentation is shown in Figure 1, and a sample of the app's response screens is shown in Figure 2.

### Measure validation

Data from three independent samples were used to evaluate the reliability and validity of the MTB measures, including Sequences: an in-person validation study in which “gold standard” tests, the NIHTB-CB Version 3 (NIHTB-CB V3), and the MTB measures were all completed in a laboratory setting (Validation Study 1); a remote validation study in which the NIHTB-CB V3 measures were administered in the laboratory, and MTB measures were completed remotely on a personal iOS or Android smartphone (Validation Study 2); and a remote test-retest study in which participants completed the MTB measures remotely twice, 2 weeks apart on a personal iOS smartphone (Validation Study 3). All samples were racially and ethnically diverse and represented a variety of educational backgrounds and age groups (Table 1). Between June and September 2021, a third-party market research company recruited participants from the general population to participate in a larger NIHTB-CB V3 norming study. Validation Studies 1 and 2 were conducted in parallel with this larger NIHTB-CB V3 renorming study. Participants in Study 3 were enrolled in a larger independent validation study and were recruited from the Brain Health Registry, which is an online longitudinal platform with over 100,000 members (Weiner et al., 2023). All participants provided informed consent prior to data collection and were compensated for their participation upon study completion. All studies were approved by the relevant Institutional Review Boards. Analyses for all validation studies were conducted in R (R Core Team, 2023).

**Table 1:** Demographic characteristics of the validation study samples.

	**Study 1**	**Study 2**	**Study 3**
	**(*N* = 92)**	**(*N* = 1,021)**	**(*N* = 168)**
	**% (*n*)**	**% (*n*)**	**% (*n*)**
**Age**			
18–29	19.57 (18)	35.75 (365)	0.60 (1)
30–39	13.04 (12)	11.66 (119)	3.57 (6)
40–49	11.96 (11)	12.83 (131)	8.33 (14)
50–59	22.83 (21)	12.24 (125)	16.07 (27)
60–69	19.57 (18)	10.87 (111)	36.90 (62)
70–79	10.87 (10)	9.30 (95)	27.98 (47)
≥80	2.17 (2)	7.35 (75)	6.55 (11)
Mean (SD)	49.27 (17.65)	43.97 (21.24)	63.54 (12.10)
Range	[20, 84]	[18, 90]	[28, 87]
**Device type**			
iPhone	100.00 (92)	63.66 (650)	100.00 (168)
Android	0.00 (0)	36.34 (371)	0.00 (0)
**Sex**			
Female	67.39 (62)	55.63 (568)	83.93 (141)
Male	32.61 (30)	44.37 (453)	16.07 (27)
Other	0.00 (0)	0.00 (0)	0.00 (0)
Not identified	0.00 (0)	0.00 (0)	0.00 (0)
**Racial identity**			
White or Caucasian	52.17 (48)	73.65 (752)	88.69 (149)
Black or African American	32.61 (30)	13.91 (142)	4.17 (7)
Asian	9.78 (9)	6.27 (64)	2.98 (5)
Native American or Alaska Native	0.00 (0)	0.69 (7)	0.60 (1)
Native Hawaiian or Other Pacific Islander	1.09 (1)	0.49 (5)	0.00 (0)
Middle Eastern or North African	0.00 (0)	0.88 (9)	0.00 (0)
Multiracial or more than one race	4.35 (4)	2.15 (22)	2.98 (5)
Other	0.00 (0)	0.00 (0)	0.60 (1)
Prefer not to say or not identified	0.00 (0)	1.96 (20)	0.00 (0)
**Ethnic Identity**			
Hispanic/Latino (any race)	1.09 (1)	14.69 (150)	7.14 (12)
Not Hispanic/Latino (any race)	98.91 (91)	85.31 (871)	92.86 (156)
Prefer not to say or not identified	0.00 (0)	0.00 (0)	0.00 (0)
**Education level**			
Less than HS	2.17 (2)	1.67 (17)	0.00 (0)
HS diploma or GED	54.35 (50)	32.03 (327)	0.60 (1)
Some college education	20.65 (19)	35.16 (359)	25.60 (43)
College or bachelor's degree (4-year degree)	15.22 (14)	20.27 (207)	32.74 (55)
Graduate or professional degree (any level)	7.61 (7)	10.87 (111)	41.07 (69)
Prefer not to say or not identified	0.00 (0)	0.00 (0)	0.00 (0)

#### Validation study 1

The goal of this study was to evaluate the convergent and discriminant validity of Sequences when completed in the laboratory on a study-provided iOS smartphone. A sample of 92 participants was recruited, all of whom took the completed MTB Sequences, the NIHTB-CB V3 LSWMT as a measure of convergent validity, and the NIHTB-CB V3 Picture Vocabulary Test (PVT) as a measure of discriminant validity. Moreover, a subsample of individuals completed the Letter-Number Sequencing (LNS) subtest (*n* = 78) and the Digit Span (DS) subtest (*n* = 77) from the Wechsler Adult Intelligence Scale, Fourth Edition (WAIS-IV) (Wechsler, 2008a) in the same testing session, as additional measures of external convergent validity.

The LNS and DS subtests from the WAIS-IV are well-established measures of working memory (Wechsler, 2008b; Hartman, 2009). The examiner recites a sequence of numbers and letters (LNS) or just numbers (DS), and the examinee is required to repeat them in the order presented, in ascending order, with letters first, followed by numbers (LNS), or forward or backward (DS). The length of the sequence increases with each trial, and the subtests end when the examinee fails to recall the sequence correctly on two consecutive trials of the same length. The LNS and DS subtests are widely used measures of working memory and demonstrate high reliability and strong evidence of validity (Wechsler, 2008a).

#### Validation study 2

The goal of this study was to evaluate the convergent and discriminant validity of the Sequences measure when administered remotely on a personal smartphone. A total of 1,021 participants were administered the NIHTB-CB measures in the laboratory, as in Study 1. Then, they completed MTB measures on their personal iOS or Android smartphones remotely, no more than 14 days later. MTB Sequences, NIHTB-CB LSWMT, and NIHTB-CB PVT were completed by 1,007 of these participants who were part of the larger MTB validation study.

#### Validation study 3

The goal of this study was to examine the test-retest reliability of Sequences when taken remotely on a personal smartphone. In the overall MTB test-retest study, 168 individuals completed the tests remotely twice following a 2-week delay interval. Of this sample, 147 completed the Sequences test-retest study.

#### Analyses

The primary goal of the three validation samples was to evaluate evidence for the reliability and validity of the MTB Sequences measure. To index internal consistency reliability, we calculated the split-half reliability (corrected with the Spearman-Brown formula) in Studies 1 and 2. We conducted 1,000 random permutations for splitting the data and correlated the two halves. The median and interquartile range (IQR) for these split-half reliability estimates provide an appropriate sampling distribution for the internal consistency of Sequences. We anticipated at least moderate internal consistency (*r*_*xx*_ > 0.70).

We also hypothesized small negative correlations with age, as working memory is anticipated to decrease in older adults. Furthermore, we anticipated moderate convergent validity (Spearman ρ > 0.50), consistent with convergent validity coefficients for LSWMT (Tulsky et al., 2014). In Studies 1 and 2, we correlated Sequences with NIHTB LSWMT, and in Study 1 only, we correlated it with the WAIS-IV Digit Span and Letter-Number Sequencing raw scores. Furthermore, we hypothesized a smaller discriminant validity coefficient when correlating Sequences with NIHTB PVT in Studies 1 and 2 compared to the correlation between Sequences and LSWMT. All correlational analyses employed Spearman's ρ correlations.

In Study 2, data were collected from both iOS and Android operating systems. Therefore, as a sensitivity analysis, we re-ran all internal consistency and correlational analyses for each operating system. We also compared the overall score distributions to determine if performance varied by operating system. Due to demographic differences between iOS and Android users, these analyses controlled for age. Furthermore, we also compared performance on Sequences based on sex assigned at birth and educational attainment (four levels: high school or less; some college education but no degree or an associates or technical degree; bachelor's degree; or advanced degree), controlling for age and with *post-hoc* pairwise comparisons adjusted using the false discovery rate (Benjamini, 2005).

Finally, we examined test-retest reliability and practice effects after a 2-week delay in Study 3. We fit a random intercept mixed-effect model with a fixed effect for study visit (0 = baseline, 1 = retest), predicting the Sequences score. The ratio of random intercept variance to total variance is an intraclass correlation coefficient (ICC) representing test-retest reliability, and the fixed effect represents practice effects. We hypothesized moderate test-retest reliability (ICC > 0.60) (Koo and Li, 2016), given that both assessment occasions occurred remotely (and uncontrolled, therefore potentially in different locations), which would also likely diminish practice effects, so they were hypothesized to be small (raw score increase of < 3 items—i.e., still within the same overall sequence length).

## Results

### Study 1

The average score for the in-person, unproctored sample was 11.2, with a median longest correct sequence length of 5. Sequences correlated 0.64 with LSWMT, 0.52 with WAIS-IV Letter-Number Sequencing, and 0.58 with WAIS-IV Digit Span. Sequences showed a notably smaller correlation with PVT (ρ = 0.34) compared to convergent measures. Table 2 summarizes the reliability and validity evidence in this (and all) samples.

**Table 2:** Reliability and validity evidence.

	**Study 1 (iOS)**	**Study 2 overall**	**Study 2 (iOS)**	**Study 2 (android)**	**Study 3 (iOS)**
Sequences raw score	*N* = 92, Mean = 11.2, SD = 4.7	*N* = 1,007, Mean = 11.9, SD = 5.0	*N* = 647, Mean = 12.2, SD = 4.7	*N* = 360, Mean = 11.3, SD = 5.4	*N* = 147 Baseline Mean = 12.7 Baseline SD = 4.0 Follow-up Mean = 13.6, Follow-up SD = 4.3
Split-half reliability median [IQR]	0.899 [0.884, 0.912]	0.903 [0.889, 0.912]	0.892 [0.878, 0.901]	0.917 [0.904, 0.926]	
Test-retest reliability ICC					0.55
Two-week practice effect					Raw = 0.91 SMD = 0.32
Corr. with age	*N* = 92, *ρ =* −0.07 *p* = 0.48	*N* = 1007, *ρ =* −0.19	*N* = 647, *ρ =* −0.13	*N* = 360, *ρ =* −0.23	
Corr. with LSWMT	*N* = 92, *ρ = 0.6*4	*N* = 996, *ρ = 0.4*6	*N* = 638, *ρ = 0.4*2	*N* = 358, *ρ = 0.5*0	
Corr. with PVT	*N* = 92, *ρ = 0.3*4	*N* = 1,000, *ρ = 0.1*8	*N* = 642, *ρ = 0.1*4	*N* = 358, *ρ = 0.2*7	
Corr. with WAIS-IV Digit Span	*N* = 77, *ρ = 0.5*8				
Corr. with WAIS-IV Letter Number Sequencing	*N* = 78, *ρ = 0.5*2				

All correlation values are significant at p < 0.001 unless otherwise noted.

Split-half reliability was high in this sample (median *r*_*xx*_ = 0.90, IQR 0.88–0.91). The relationship between MTB Sequences and external validity measures was also strong and consistent with our hypotheses, especially for NIHTB LSWMT. Surprisingly, Sequences was only minimally related to chronological age.

### Study 2

The average score for the remote sample was 11.9, with a median longest correct sequence length of 5. Split-half reliability was high in the total sample (median *r*_*xx*_ = 0.90, IQR 0.89–0.91). The relationship between Sequences and LSWMT was moderate (ρ = 0.46) and somewhat lower than that observed in Study 1. However, the relationship with age in this larger sample was more consistent with our hypotheses, showing a greater decrease in ability with age (ρ = −0.19) compared to Study 1. Discriminant validity comparison between Sequences and PVT was lower than in Study 1 (ρ = 0.18), and it was again meaningfully smaller than the convergent validity comparison.

A total of 647 individuals completed Sequences remotely on an iOS device, while 360 completed it on an Android device as part of Study 2. Comparing performance across devices initially suggested that Android users scored lower (worse) than iPhone users [difference = −0.94, *F*_(1, 1, 005)_ = 8.49, *p* = 0.003], but there were significant age differences between the samples as well, which likely confounded this relationship. After adjusting for age, the difference in performance by operating system was no longer significant [expected mean difference = −0.42, *t*_(1, 004)_ = −1.23, *p* = 0.22, ES = −0.085]. See Table 3 for unadjusted and adjusted means and associated comparisons.

**Table 3:** Comparisons by demographic characteristics in sample 2.

	***N***	**Mean**	**SD**	**Unadjusted comparison**	**Age-adjusted marginal mean**	**Adjusted comparisons**
Android	360	11.3	5.4	Android < iOS; *p* = 0.003; ES = 0.19	11.6	Nonsignificant; *p* = 0.22; ES = 0.085
iOS	647	12.2	4.7		12.0	
Male	448	11.7	5.2	Nonsignificant; *p* = 0.31; ES = 0.06	11.6	Nonsignificant; *p* = 0.17; ES = 0.09
Female	559	12.0	4.8		12.1	
High School or Less (a)	341	11.3	4.8	a < c; *p* = 0.02; ES = 0.27 a & b, a & d, b & c, b & d, c & d nonsignificant; *p* > 0.05; all ES < 0.16	11.2	a < c; *p* = 0.001; ES = 0.33 a < d; *p* = 0.03; ES = 0.27 b < c; *p* = 0.03; ES = 0.23 a & b, b & d, and c & d nonsignificant; *p* > 0.05; all ES < 0.16
Some College Education (b)	354	11.9	5.0		11.7	
Bachelor's Degree (c)	204	12.6	5.1		12.8	
Advanced Degree (d)	108	12.1	5.1		12.5	

The false discovery rate was used to control for multiple comparisons across educational attainment levels.

Across the two operating systems, the psychometric properties were also broadly consistent. Split-half reliability was high in both samples (median *r*_*xx*_ = 0.89 and 0.92), and the relationship with LSWMT was also acceptable (ρ = 0.42 and 0.50), though it was lower than the convergent validity correlation observed in Study 1 (where ρ = 0.64).

Within this sample, we also compared performance on Sequences by sex assigned at birth and educational attainment, as shown in Table 3. There was no difference by sex assigned at birth [*F*_(1, 1, 004)_ = 1.07, *p* = 0.30; effect size (ES) = 0.09]. There were significant differences found across educational levels [*F*_(3, 1, 002)_ = 3.27, *p* = 0.02]. Broadly, individuals with high school education or less performed worse than college graduates, and those with some college education performed worse than those with a bachelor's degree. Although those with an advanced degree were slightly lower than those with a bachelor's degree, this was not a statistically significant difference.

### Study 3

Of the 168 individuals who enrolled in the test-retest study and completed at least one MTB measure at baseline, 147 successfully completed Sequences twice within 14 ± 3 days on an iOS device. The average score for the remote sample was 12.7, with a median longest correct sequence length of 5. Test-retest reliability was moderate (ICC = 0.55). Individuals did improve at the second assessment, but as hypothesized, they were still largely succeeding at the same sequence lengths. The average improvement in scores was less than one additional item correctly recalled. However, due to variation in the sample, the standardized mean difference in performance was moderate (SMD = 0.32).

## Discussion

We describe the development and validation of Sequences, an innovative measure of working memory that can be self-administered on a participant's smartphone.

Convergent validity of Sequences was evaluated in two separate studies—one in which Sequences was administered unproctored but in a monitored testing room (Study 1) and one in which participants completed the measure remotely within 14 days of being administered the target convergent measures (Study 2). In Study 1, Sequences showed moderately strong correlations to working memory measures on the NIHTB-CB (LSWMT) and the WAIS-IV (LNS and DS), all of which exceeded the hypothesized values. Moreover, the correlations were similar in magnitude to those observed in the original validation study of LSWMT (Tulsky et al., 2014). In Study 2, Sequences showed an acceptable correlation with LSWMT, but it was notably lower than for Study 1 (ρ = 0.46 vs. ρ = 0.64). The convergent correlations were slightly higher for users of Android devices, but the difference was not significant. As hypothesized, discriminant validity correlations with NIHTB-CB PVT in Studies 1 and 2 were lower than the convergent validity correlations, with the correlations between measures being lower in Study 2 than Study 1.

The remote administration of Sequences in Study 2 compared to in-person administration of the external measures may have contributed to the lower convergent validity correlation observed. In a remote self-administration setting, there is no control over the study location or background noise, no assurance that participants remain consistently attentive to the task, and no control over participant dishonesty (e.g., writing down the stimuli as presented, which undermines any working memory mechanisms). Moreover, a delay of up to 14 days between completing the external measures and the Sequences measure may introduce other sources of error that can impact the observed correlations. Despite these factors, the moderate correlation observed between Sequences and LSWMT is relatively in line with expectations for convergent validity.

Based on existing research on the trajectory of working memory throughout the lifespan, we anticipated that performance on Sequences would peak in early adulthood and then decline with age (Bopp and Verhaeghen, 2005; Van der Linden et al., 1994; Grégoire and Van Der Linden, 1997). In Study 1, a minimal, non-significant, negative association between age and performance was observed (ρ = −0.07), while Study 2 showed a small negative correlation (ρ = −0.19). While Study 1's sample size was too small to evaluate any meaningful age-related effects, the results from Study 2 suggest that performance on Sequences changes with age in the expected direction, albeit with a weaker relationship than expected. Nevertheless, age was found to be more strongly associated with performance than education, sex assigned at birth, or the operating system used.

The results provided strong evidence of the internal consistency reliability of Sequences, with split-half correlations of 0.90 in both Study 1 and Study 2. Test-retest reliability showed a moderate correlation after a 14-day delay, slightly below but similar to the hypothesized value. We would have hypothesized a much higher correlation had testing been conducted in a controlled environment, as is typical of such measure evaluations; however, since participants might have completed the two Sequences self-administrations in completely different environments and with different levels of attention, a lower retest correlation may be indicative of real-world conditions and underscores the importance of analyzing group results rather than individual outcomes.

All cognitive tests that are self-administered remotely have inherent challenges, given the variability in testing environment (including noise and distractions), screen size, participant effort and attention levels, and possible dishonesty. Despite these inherent obstacles, Sequences shows evidence of an reassuring level of robustness, even when compared to “gold standard” measures of a similar construct. In addition, there was evidence of adequate floor and ceiling effects for the measure across a broad range of ages, and the median administration time met expectations (5 min).

### Limitations

As noted, only Study 2 included Android and iOS users, so any inferences about the effect (or lack thereof) of the operating system on participant performance must be interpreted with caution. Further research replicating Studies 1 and 3 to confirm our findings would be a valuable addition to the literature. Furthermore, a larger sample could evaluate other hardware and software differences, such as screen size and device type, which were beyond the scope of this study's data collection efforts. In addition, a replication of Studies 2 and 3 would benefit from information on the remote context in which users completed each measure. This information was not available for the present study but would provide a valuable source of data for future research efforts.

## Conclusion

Sequences is a self-administered, smartphone-based working memory measure within the Mobile Toolbox that was developed *de novo* to match a measured NIH Toolbox construct while working within the constraints of remote self-administration. Our findings provide evidence to support the reliability and validity of Sequences when self-administered on a personal smartphone. Future versions of the measure should be developed to monitor and control for respondent dishonesty in completely unsupervised environments as much as possible. While it is crucial to evaluate aggregated results rather than individual outcomes when using self-administered tests of cognitive functioning, findings suggest that Sequences serves a valid measure of working memory in research studies for adults throughout their lifespan.

## Data Availability

The datasets presented in this study can be found in online repositories. The names of the repository/repositories and accession number(s) can be found at: doi: 10.7303/syn52229757.
