# Supplementary material for: Mobile Toolbox sequences task: development and validation of a remote, smartphone-based working memory test
Source: Front Psychol. 2025 Jan 22;15:1497816. doi: 10.3389/fpsyg.2024.1497816 (PMC11794243; doi:10.3389/fpsyg.2024.1497816)
Supplement: Supplementary file 1 [file Data_Sheet_1.PDF]

Supplementary Online Material for:

Mobile Toolbox Sequences Task: Development and Validation of a Remote, Smartphone-Based Working Memory Test

Pilot Testing Methodology

Prior to using Sequences in a larger population, we conducted pilot studies to understand the feasibility of self-administration on a personal smartphone in a remote context. Specific research questions and sample size estimates to address feasibility and usability concerns were derived as part of this process. All pilot tests were conducted using experimental versions of the measure on iOS devices.

Based on qualitative feedback from our UI/UX expert and the default layout of keyboards varying across devices, it was determined that an app-specific button layout would be necessary. Mis-keyed entries were too common with the native keyboard. Further, keyboards vary across phones and operating systems, such that the numbers may or may not be above the letters; having to toggle back and forth between a number pad and letter pad would reduce feasibility and result in negative user interactions. Therefore, we created a custom display with both letters and numbers on the same screen. Moreover, having a limited display allowed for larger buttons than the native keyboard would have enabled, thereby mitigating (but not eliminating) the concern of mis-keying responses.

Two separate pilot studies were conducted to aid the development of Sequences. An alpha version (or in the case of competing models, two versions) of Sequences was programmed within the Mobile Toolbox (MTB) app. A convenience sample of participants, who were identified through a market research firm, downloaded the MTB app from the iOS app store,

preloaded with only the relevant Sequences task, and used their study-assigned ID to access the measure. Sampling targets for racial and ethnic groups, educational attainment, and age were specified for each pilot study to have adequate representation of meaningful participant characteristics, but insofar as the emphasis of these pilot tests was on the feasibility of the measure and not on an individual's performance, participant-specific demographics were not closely analyzed for these pilot studies.

Pilot Study 1

In Pilot Study 1, two distinct forms (A and B) were administered to evaluate how examinees responded to two sets of letters, given that only 9 could be displayed as response options. Form A had 24 scored items (3 each at sequence lengths 3-10) using the numbers 1-9 and the letters A-B-C-D-E-F-G-H-J, and form B had 24 distinct items with the same sequence lengths, but using the letters A-B-C-J-K-L-X-Y-Z. Each form began with two sample items (to clarify instruction, but with no feedback provided to participants), and items were administered by the app one at a time, until a participant gave incorrect responses to all 3 items at a given sequence length. Each letter or number was displayed for 1 second in each sequence. Seventy-five participants completed Form A and 74 completed Form B ($n = 149$) remotely. Participants ranged in age from 18-63.

Results from Pilot Study 1 showed that 18 Form A respondents (24.0%) and 21 Form B respondents (28.4%) failed to get any items correct, suggesting significant floor effects. One participant obtained a perfect score on Form B, which was unexpected but not of sufficient frequency to be considered a problematic ceiling effect. There were no significant differences between pilot versions, suggesting equal difficulty for the two sets of letters. The means for

Form A was 6.5 (SD = 5.6) and for Form B was 7.8 (SD = 6.5), which were not significantly different from each other (t(143.15) = -1.27, $p = 0.21$).

Pilot Study 2

Because of the significant floor effects observed in Pilot Study 1, we raised feasibility questions and concerns with our UI/UX expert (as described in the main paper). The qualitative feedback suggested that the two practice items alone were insufficient for individuals to understand the test. Further, the expert noted that best practices in stimulus presentation on mobile devices suggest utilization of lowercase letters.

Based on the aggregation of qualitative and quantitative information gleaned from Pilot Study 1 and the UI/UX input, the following changes were made for Pilot Study 2 to ameliorate floor effects and improve test usability: 1) corrective feedback for practice trials and one additional instruction screen were added to the task, to ensure participants understood how to complete it; 2) three new items were added with a sequence length of 2 and a second set of three items was added with a sequence length of 3, to better differentiate among individuals of lower ability on the task and to lower the test floor; 3) lowercase letters, grouped in distinct sets of three rather than being continuous, were preferred, and so the new set utilized was a-b-c-q-r-s-x-y-z.

The primary goal for Pilot Study 2 was to evaluate whether the changes mitigated floor effects on the Sequences task. As such, only one form was used. The maximum number of scored items was 30, with all participants starting with a sequence length of 2 (to avoid reversals and resultant confusion in a self-administered context). The same discontinue rules were applied as in Pilot Study 1—that is, all three trials within a set at a given sequence length had to be

incorrect to terminate the test. The same market research firm was employed to recruit an independent sample of participants for Pilot Study 2 (n = 144; age range 18-85).

Participants completed the second pilot remotely on an iOS device. Only four respondents (2.8%) obtained a zero score, and no participant obtained a perfect score. Scores were well distributed overall (see Figure S1). Median test administration time was approximately 5 minutes. Based on these results, we concluded that the previously observed floor effects had been adequately mitigated, and that Sequences was ready to proceed with the larger validation study described in the main manuscript.

Figure S1: Score distribution from Pilot Study 2

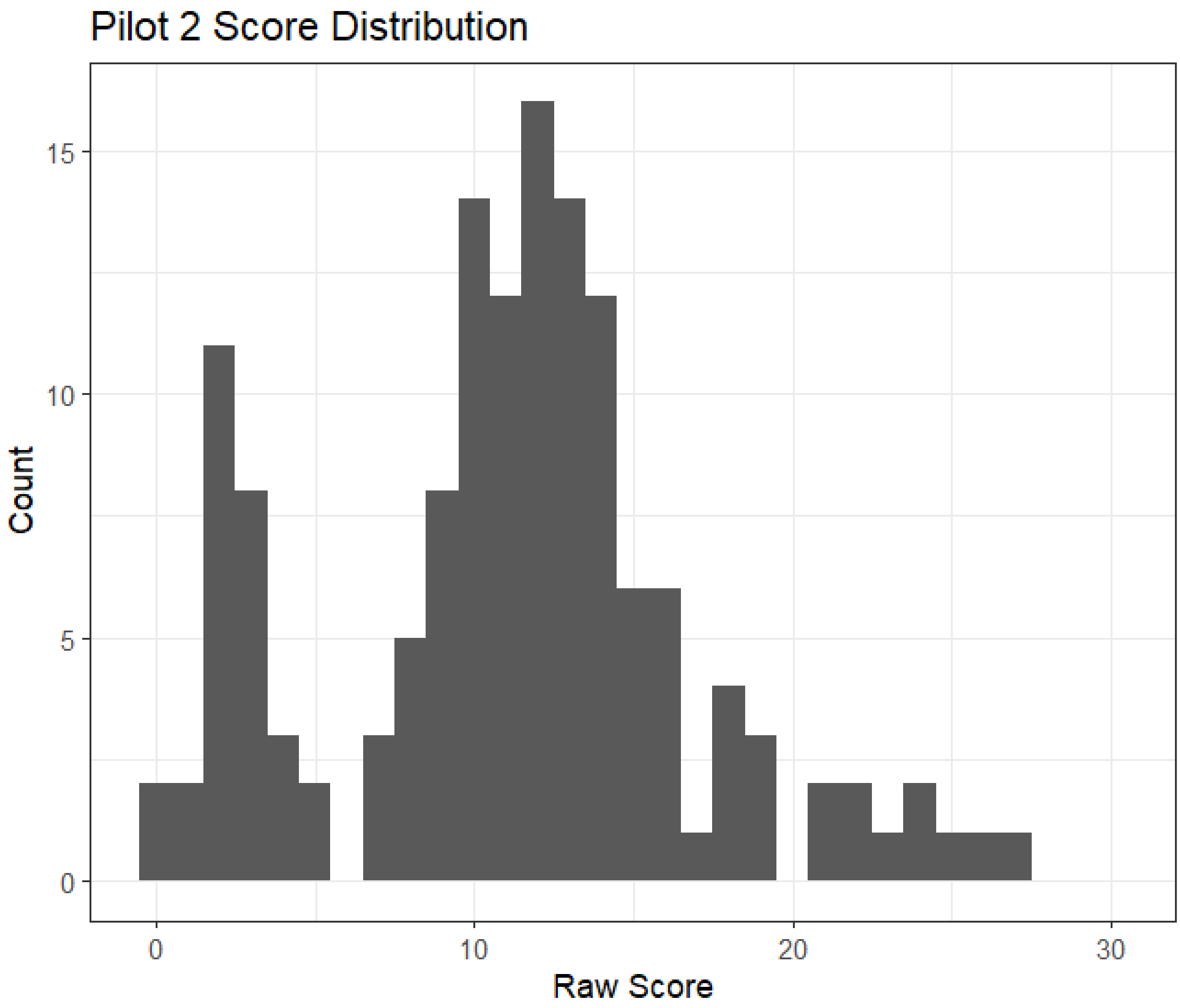

Note: the possible score range was 0 to 30. The observed scores ranged from 0 to 27. The median number correct was 12 (mean = 11.2, SD = 5.7).
